# Incidence and Mortality of Spontaneous Subarachnoid Hemorrhage in Martinique

**DOI:** 10.1371/journal.pone.0155945

**Published:** 2016-05-23

**Authors:** Mathieu Schertz, Hossein Mehdaoui, Abderrahmane Hamlat, Michel Piotin, Rishika Banydeen, Mehdi Mejdoubi

**Affiliations:** 1 Department of Neuroradiology, University Hospital of Martinique, Fort de France, Martinique (FWI); 2 Department of Critical Care and Emergency, University Hospital of Martinique, Fort de France, Martinique (FWI); 3 Department of Neurosurgery, University Hospital of Martinique, Fort de France, Martinique (FWI); 4 Department of Interventional Neuroradiology, Rothschild Foundation, Paris, France; 5 Clinical Research Department, University Hospital of Martinique, Fort de France, Martinique (FWI); Heinrich-Heine University, GERMANY

## Abstract

**Background:**

Incidence of spontaneous subarachnoid hemorrhages (SAH) varies wildly across the world and seems to be low in Central and South America (4.2 per 100 000 person-years; CI 95%; 3.1–5.7). The objective of our study was to describe the characteristics of SAH and to estimate its incidence and severity in Martinique, a small French island located in the Caribbean Sea.

**Methods:**

Due to its insular nature and small captive population, Martinique is ideal for the setting up of population-based epidemiological studies with good exhaustiveness. Our study, spanning a 7 year period (2007–2013), consisted of retrospective case ascertainment with multiple overlapping methods. Crude incidence and 30 day case-fatality rates for SAH among the Martinican population were computed for the study period. Incidence and disease severity was also analyzed according to age, gender and aneurysm presence. World age-standardized incidence rates were also calculated.

**Results:**

A total of 121 patients had a SAH during the study period, with a higher frequency of female cases (71.1% versus 28.9%, p<0.001). Patient mean age was 57.1 years (median = 55 [46–66]). An aneurysmal origin was found in 96 SAH cases (79.3%). Crude annual incidence was 4.36 per 100 000 person-years (CI 95% 2.30–6.42). World age-standardized incidence was 3.29 per 100 000 person-years (CI 95% 1.74–4.84). During the 30 days following SAH diagnosis, 29 patients died (case fatality rate: 24% (CI 95% 16.4–31.6)).

**Conclusions:**

The incidence of spontaneous subarachnoid hemorrhage in Martinique is much lower than in other parts of the world and similar to countries in Central and South America. These results are possibly related to environmental factors and most particularly to a low rate of smoking in the Martinican population. Thirty-day case-fatality rate is similar to what is observed in developed countries.

## Introduction

The worldwide incidence rate of spontaneous subarachnoid hemorrhage (SAH) is approximately 9.1 per 100 000 person-years but varies widely between countries. Incidences are much higher in Finland (19.7 per 100 000 person-years 95% CI; 18.1–21.3) and Japan (22.7 per 100 000 person-years CI 95%; 21.9–23.5) and lower in South and Central America (4.2 per 100 000 person-years; CI 95%; 3.1–5.7)[1]. With a 30-day case fatality of 35% to 45%, subarachnoid hemorrhage is the most deadly and debilitating subtype of stroke. Similarly to incidence, case-fatalities for SAH also show potentially important regional differences [2]. While incidence disparities around the world can be related to genetic and environmental factors, differences in the disease’s severity are linked to quality of and access to healthcare.

Ruptured intracranial aneurysm is the most common cause of spontaneous SAH (around 85% of cases) [3]. Physiology of aneurysm formation and rupture is however still not fully understood and epidemiological studies on SAH could help us further understand the natural history of the disease as well as better identify risk and prognostic factors that could lead to markers of disease mechanism [4]. Moreover, there is a prevailing lack of information about SAH incidence and outcome in some parts of the world, more particularly in populations of African descent [5].

The objective of this study is to describe the characteristics of SAH and to estimate its incidence and severity in Martinique, a small French overseas island with a population of mostly African descent [6].

## Patients and Methods

### Area of Investigation

Martinique is a French overseas island located in the south of the Caribbean basin (latitude 14°30 North, longitude 61° West). Its surface area is 1128 km^2^ and its climate is tropical, with an annual average temperature ranging from 22°C to 30°C [7]. In 2008, Martinique was classified as a very high human development territory with a human development index of 0.904 [8]. As such, it is currently considered one of the most developed islands of the Caribbean region. The island is also densely populated (356 persons/km^2^) with a total of 395 027 inhabitants in 2012 [9]. The latter are mostly French African Caribbeans, descendants of black Africans, French Caucasians, and immigrants from the Indian subcontinent. The population is served by 12 public hospitals, including the University Hospital of Martinique, 1 small private hospital, 291 general practitioners (GPs), 2 private neurologists, and 2 on-call physician systems [7].

Because of its small geographical size, insular nature and captive population, Martinique island represents an ideal setting to study SAH incidence and fatality. Medical care is free of charge, allowing unrestricted access to hospitals, high-quality medical services and diagnostic technologies, including CT (computerized tomography) and MRI (Magnetic Resonance Imaging). Moreover, on the island, a single interdisciplinary team of experienced radiological, neurosurgical and neurological experts is handling all patients with clinically suspected cerebrovascular accidents at the University Hospital of Martinique (the study center). Consequently, a minimal number of SAH cases were missed, thus guaranteeing a fairly good exhaustiveness in terms of case ascertainment for this study.

### Case ascertainment

All cases of stroke suspicion in Martinique are referred to the University Hospital of Martinique. Our retrospective study consisted in identifying all stroke suspicions reported between January 1^st^ 2007 and December 31^st^ 2013.

For case ascertainment over the 7-year study period, multiple overlapping methods were employed: (1) the hospital’s administrative patient databases were searched for all patients coded with the ICD-10 code (International Classification of Disease, 10^th^ Revision) defining non-traumatic SAH and/or having a discharge diagnosis consistent with subarachnoid hemorrhage (I60, I61.5, I61.9, I62, I67.1, I69, I72.0, I72.9) [10]; (2) the imaging database at our institution was searched for all patients undergoing cerebral angiography, CT-scan and MRI by screening the database with the keywords “aneurysm”, “Subarachnoid Hemorrhage”and other synonyms in the French language; (3) the hospital’s surgical database was searched for all patients undergoing aneurysm surgery; (4) the emergency medical department’s database was searched for all patients registered as having a SAH.

It is to be noted that patients having died before hospital admission or before receiving medical attention could not be considered. In Martinique, necropsy is very rarely performed because of local tradition. Consequently, autopsy diagnosis of SAH, neuroimaging of dead patients and resulting patient death certificates could not be evaluated as a potential source of declaration for SAH for our study.

For all identified patients, medical records and imaging tests were reviewed. A diagnosis of subarachnoid hemorrhage was accepted if there was a history of sudden onset of severe headache or unconsciousness with signs of meningeal irritation with or without focal neurological deficit, supported by CT, MRI or lumbar puncture evidence of subarachnoid hemorrhage (xantochromia detected by visual and/or spectrophotometric examination).

Careful elimination of all duplicate entries based on last name and date of birth was performed.

Patients were only included in the study if they presented with their first ever subarachnoid hemorrhage. Patients having an intra-cerebral hemorrhage, associated with SAH, were included only if the origin of the bleeding was clearly identified as an intracranial aneurysm. Patients who were not residents of Martinique, patients with a primary history of head trauma, patients having arteriovenous or other malformations were excluded.

### Study data

All study data were recorded with the approval of the University Hospital of Martinique’s ethics committee (Comité de Protection des Personnes Sud-Ouest et Outre Mer III).

Outcome data corresponding to the patient’s vital status were obtained from medical records and when necessary, general practitioners of study patients were contacted for further information. Death within the 30 days following SAH diagnosis, regardless of cause, was considered for case-fatality computations. The following information was also noted: gender, age, city of residence, diagnostic modality, presence of an aneurysm and its location on the circle of Willis (if multiple aneurysms were present, only the location of the aneurysm supposed to be responsible for SAH was registered).

Particular attention was paid during case diagnosis of perimesencephalic hemorrhage (defined as hemorrhage restricted to the cisterns surrounding the brainstem and suprasellar cistern, coupled to a negative cerebral angiogram). These cases were included for the study’s purpose but analyzed separately.

### Statistical Analysis

Patient information was anonymized prior to analysis.

The number of SAH episodes was recorded each year from January 1^st^ 2007 to December 31^st^ 2013. Incidence rates were presented as SAH episodes per 100,000 person-years with a 95% confidence interval (CI). Population denominator data was obtained from yearly population estimates issued by the French National Institute for Statistics and Economical Research [9]. Overall crude incidence rate, as well as SAH incidence according to age, gender and aneurysm presence, were calculated. Age standardized incidence rates were also computed by direct standardization using the World Health Organization’s World Standard Population for 2000–2025 [11]. Overall and sex-specific 30-day case-fatality rates were also generated by considering deaths, regardless of cause, among the study patients. Confidence intervals were generated by assuming a Poisson distribution for all case counts.

For all descriptive and inferential analyzes, assumption of normal distribution of the data was analyzed by the Shapiro and Wilk test as well as by the kurtosis and skewness standardized coefficients. Underlying assumptions of each statistical procedure were tested, and the following tests were consequently used for group comparisons: Student t-test, Wilcoxon-Mann-Whitney test, Chi-square test and Fisher’s exact test. Mean and 95% confidence intervals were reported for normally distributed variables and median and interquartile range for non-normally distributed variables.

All statistical analyses were conducted using SPSS software 22.1 for Windows (SPSS, Inc, Chicago, IL). All inferential analyses were performed by means of a 2-sided test, with a level of significance of 5%.

## Results

**Fig 1:**
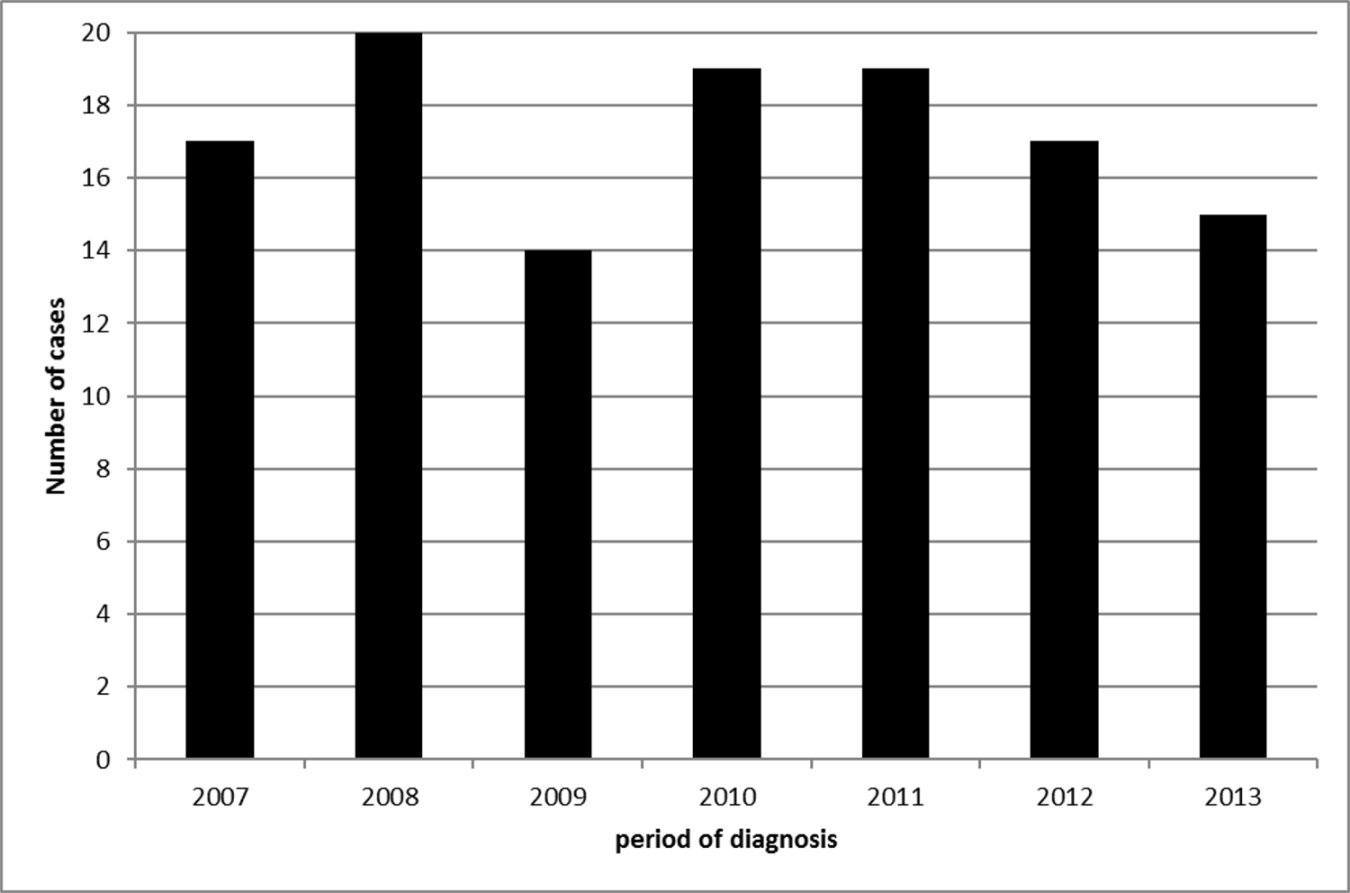
Number of spontaneous subarachnoid hemorrhages (SAH) recorded annually in Martinique between January 1st 2007 and December 31st 2013.

From 2007 to 2013, there were 121 episodes of SAH in Martinique. There was on average 17 SAH cases annually, with a low variability in SAH occurrence during the 7-year study period (Fig 1). Crude SAH incidence was 4.36 per 100 000 person-years (CI 95% 2.30–6.42). World age-standardized SAH incidence was 3.29 per 100 000 person-years (CI 95% 1.74–4.84). Overall, incidence was found to be twice as high in women (4.24 per 100 000 person-years (CI 95% 1.87–6.62)) as compared to men (2.09 per 100 000 person-years (CI 95% 0.26–3.26)).

The general characteristics of the SAH cases are resumed in Table 1. A significantly higher frequency of female patients was observed (71.1% versus 28.9%, p<0.001). The mean age at diagnosis was 57.1 years (median = 55 [46–66]). The youngest patient was aged 20 years while the oldest was 95 years. The mean age for women was 57.3 years (median = 55 [44–72]) as compared to 56.6 years (median = 54 [51–63]) for men.

**Table 1:** General characteristics of patients diagnosed with spontaneous subarachnoid hemorrhages (SAH) in Martinique between January 1st 2007 and December 31st 2013.

	Total	Women	Men	P-value^α^
**Number of SAH (%)**	121	86 (71.1%)	35 (28.9%)	<0.001
**Median age (years)[Interquartile range]**	55 [46–66]	55 [44–72]	54 [51–63]	NS
**Number of aneurysmal SAH (%)**	96 (79.3%)	68 (79.1%)	28 (80.0%)	NS
**Number of multiple aneurysms (%)**	24 (25.0%)	19 (27.9%)	5 (17.9%)	NS
**Case fatality (%)**^**b**^	29 (24.0%)(16.4–31.6)	17 (19.8%)(11.4–28.2)	12 (34.3%)(18.6–50.0)	NS

^α^ statistical significance: p<0.05.

^b^95% confidence intervals.

SAH was assessed in all cases by imaging techniques (91.7% with CT and 8.3% with MRI). Ninety-five patients (78.5%) had an additional conventional angiography. In 4 SAH cases, no vascular imaging modality (angiography, MR angiography or CT angiography) was performed.

During the study period, 96 SAH cases (79.3%) had an aneurysmal origin (median = 54 [45–56] years). Concerning the 4 SAH cases for whom no vascular imaging modality was performed, SAH was considered to be from aneurysmal origin, as the latter is the most commonly described etiological factor [3].

**Fig 2:**
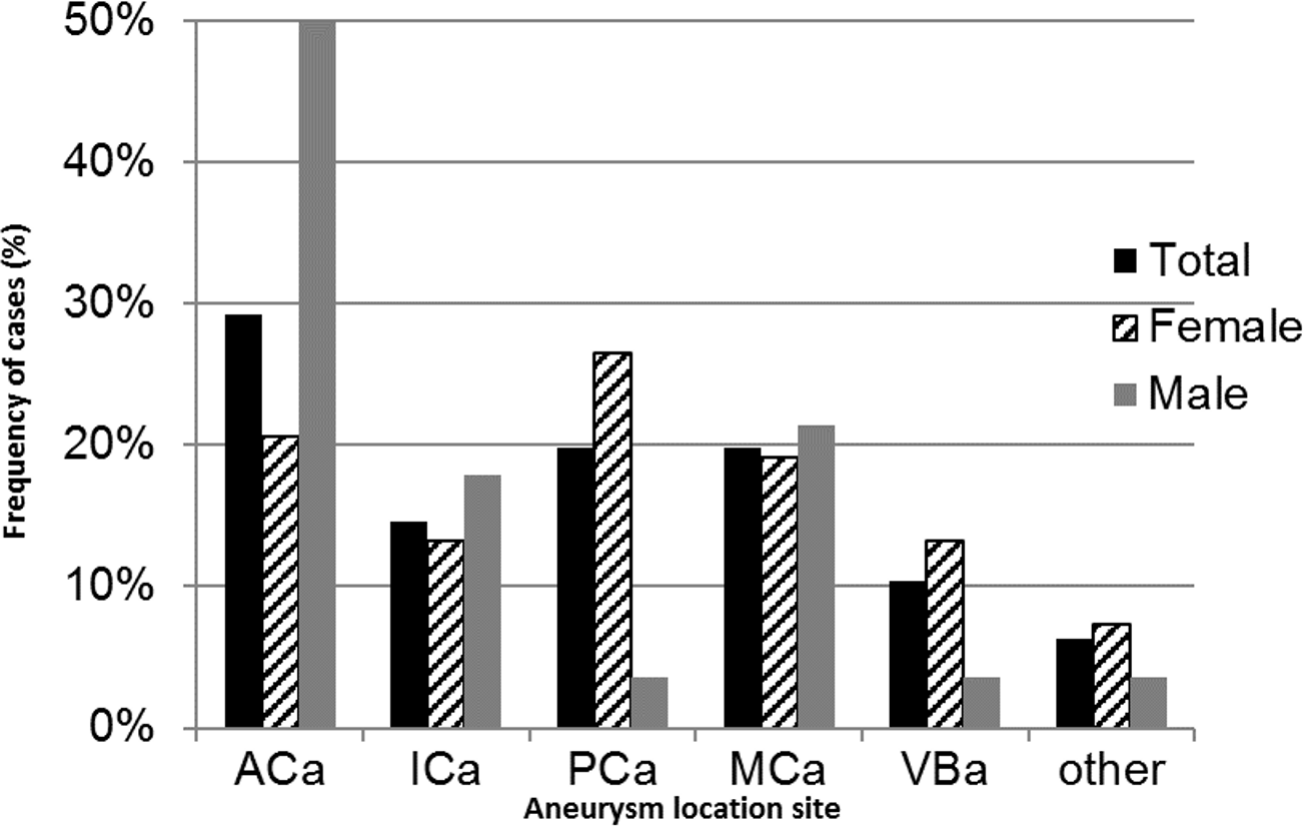
Distribution of aneurysm location, according to gender, on the circle of Willis in patients diagnosed with spontaneous subarachnoid hemorrhages (SAH) between January 1st 2007 and December 31st 2013 in Martinique. Figure legend: ACa: Anterior communicating artery, ICa: Internal carotid artery, PCa: Posterior communicating artery, MCa: Middle cerebral artery, VBa: Verterbrobasilar arterial circulation, other: peri-callosal artery and 4 cases of SAH without vascular imaging exploration.

Twenty five percent of patients harbored multiple aneurysms. The overall and sex-specific distribution of aneurysm location on the circle of Willis is shown in Fig 2. The posterior communicating artery was the most frequent aneurysmal location in women (26.5%), while for men, aneurysm was most prevalent at the level of the anterior communicating artery (50%).

Crude aneurysmal SAH incidence was 3.46 per 100 000 person-years (CI 95% 2.05–5.84). Standardized aneurysmal SAH incidence was 2.67 per 100 000 person-years (CI95% 1.41–3.94). There was also a significantly higher number of female patients diagnosed with aneurysmal SAH (70.8% versus 29.2%, p<0.001). Aneurysmal SAH incidence was higher in women (3.47 per 100 000 person-years (CI95% 1.29–5.65)) as compared to men (1.69 per 100 000 person-years (CI95% 0.03–2.75)).

During the 30 days following SAH diagnosis, 29 patients died (case fatality rate: 24% (CI 95% 16.4–31.6)). No significant difference was observed between men and women (34.3% in men versus 19.8% in women). Case fatality rate in patients with aneurysm was 27% (CI 95% 18.2–36.0) as compared to a case fatality rate of 8.3% (CI 95% 0–17.4) in patients without aneurysm.

Moreover, during the study period, 11 perimesencephalic hemorrhages (7 men and 4 women, mean age 55±16 years) were observed. All perimesencephalic hemorrhages were verified with CT angiography, among which 8 cases had an additional conventional angiography. No aneurysm was found in these patients. None of them died within 30 days.

## Discussion

**Fig 3:**
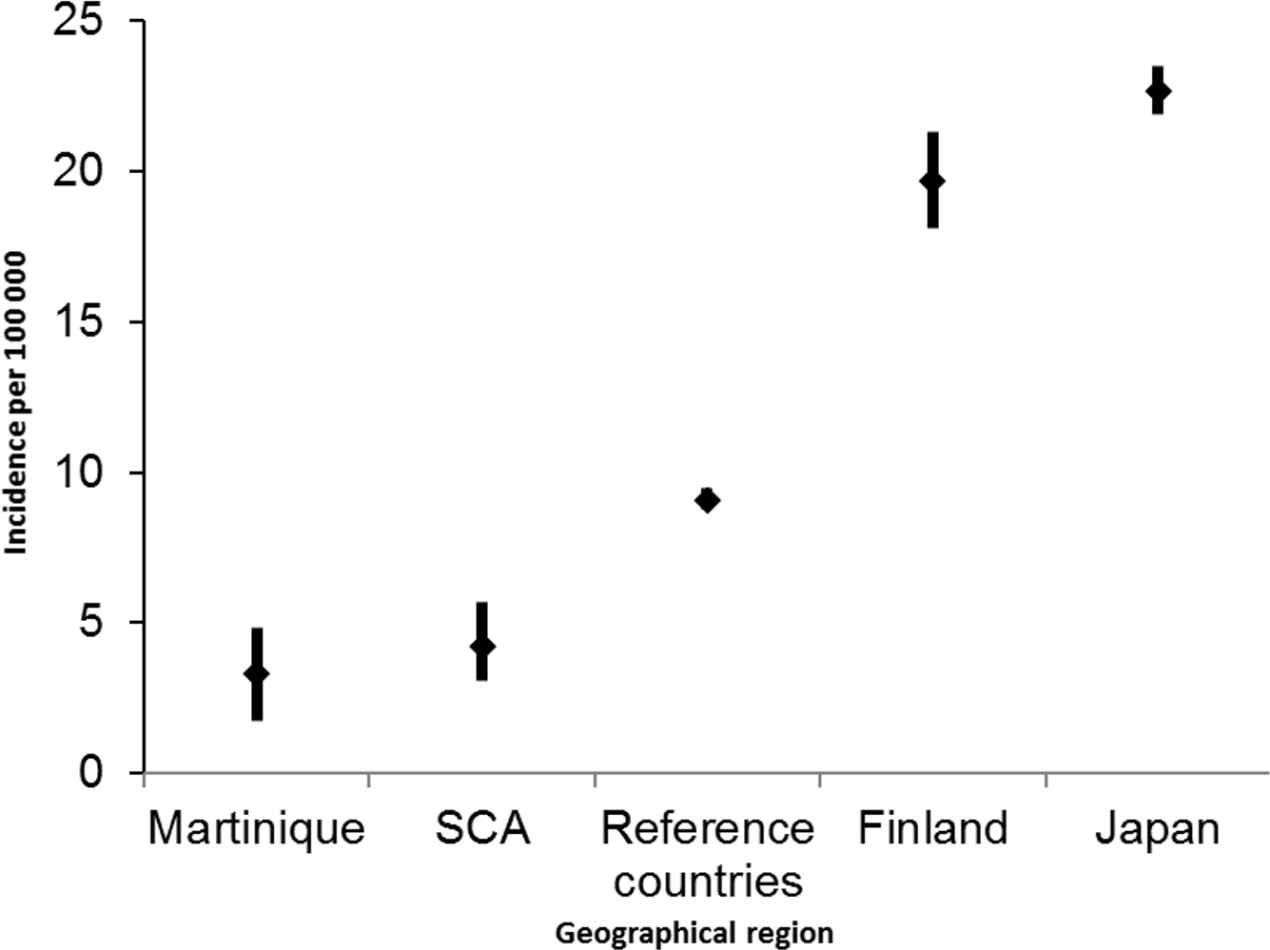
SAH incidence in Martinique compared to other regions. Figure legend: Results are compared with the meta-analysis of de Rooij *et al*. [1]. All countries other than Japan, Finland and South and Central America (SCA) were pooled in a reference group (Reference countries). Number per 100 000 person-years, with corresponding 95% CI, are represented.

Incidence of SAH is known to vary significantly between countries, with doubled rates in Japan and Finland and far lower rates in South and Central America [1]. The present study finds a world age-standardized incidence of SAH of 3.29 per 100 000 person-years (CI 95% 1.74–4.84) for Martinique island, much lower than other geographical regions represented in Fig 3. The Martinican rate remains however similar to incidence observed in the neighboring Caribbean island of Barbados (2.9 per 100 000 per years (CI 95% 1.6–6.0)) [12].

Incidence variations can be related to genetic and environmental factors [13–14]. SAH incidence similarities between Martinique and Barbados underlie these etiological hypotheses as both islands share common geographical, environmental and genetic factors. In 2007, Wolfe *et al*. compared stroke incidence of Barbadians living in South London and Barbadians living in Barbados [15]. The risk of SAH was found to be five times higher in the immigrant population of South London than in the Barbadian population (IRR, 5.04; 95% CI[2.54 to 9.97]), further suggesting a more pro-eminent role of environmental factors as compared to genetic ones.

Moreover, similarly to what is reported by de Rooj *et al*.’s systematic review, our results describe an increased risk for SAH and intracranial aneurysm in women as well as with increasing age [1]. Our overall 30-day case fatality rate of 24% (CI 95% 16.4–31.6) is rather similar to what is observed in developed countries [2].

Consistent with literature [16–17], the most common site of aneurysm was the anterior communicating artery in men and posterior communicating artery in women. During the 7-year study period, the proportion of SAH without aneurysmal origin in Martinique was 20.7% and 11 perimesencephalic hemorrhages were diagnosed. These proportions are also similar to what is reported by other authors [3, 18].

Known risk factors for SAH are smoking (risk multiplied by 3), arterial hypertension (risk multiplied by 2.8) and excessive alcohol intake (risk multiplied by 1.5) [13, 19]. Smoking and hypertension seem to have an additive effect. And, while pathogenic mechanisms leading to aneurysm formation and rupture (cause of 85% of SAH cases) are still not fully understood, smoking is further hypothesized to play an important role in aneurysmal development. As such, smoking, hypertension and alcohol intake are potential modifiable risk factors which can be acted upon in order to reduce SAH risk within a population. Recent studies [13, 20] further suggest that diabetes and obesity could have a protective role against SAH. In Martinique, the population has a high rate of arterial hypertension, diabetes and obesity [21] but tobacco-smoking rate is very low (16% for men and 5% for women) when compared to global rates of 31.1% and 6.2% for men and women respectively [22]. Unfortunately, our study’s retrospective nature does not allow us to analyze the role of these factors in observed SAH trends on the island.

Martinique however remains an ideal setting for an epidemiological study on SAH because of its small geographical size, its insularity with a captive high-density population as well as an easy unrestricted access to hospitals, quality health services and diagnostic facilities. The island also benefits from well-organized emergency ambulance services and a good awareness level regarding stroke pathology amidst the local population. Moreover, a single interdisciplinary team of radiological, neurosurgical and neurological experts handles all patients with clinically suspected cerebrovascular accidents. For our research work on SAH, all identified cases were verified by CT or MRI and a systematic review of all medical records ensured the exclusion of primary intracranial hemorrhage and subarachnoid hemorrhage secondary to other causes. All these factors, coupled to the use of a strict SAH case definition as well as multiple overlapping methods for case ascertainment (4 information sources), guarantee minimal bias in the reporting of SAH cases as well as a good reliability of our results concerning SAH incidence and fatality. This assumption is further consolidated by the results of the one-year prospective population-based study on stroke incidence, ERMANCIA II which was conducted from November 1^st^ 2011 to October 31^st^ 2012 in Martinique by Olindo *et al* [7]. With our study methodology, we found the exact same number of patients with SAH (17 patients) over the same time period as ERMANCIA II.

However, the study’s retrospective nature and the current state of medical procedures in Martinique did not allow us to consider those patients who died before hospital admission or before receiving medical attention. While we do know that between 10 and 15% of SAH die before reaching hospitals [3], necropsy is very rarely available because of local tradition. Consequently, autopsy diagnosis of SAH, neuroimaging of dead patients and resulting patient death certificates could not be evaluated as a potential source of declaration for SAH for our study. This limitation may underestimate the rate of SAH in Martinique but autopsy or neuroimaging are rarely performed in most epidemiological studies on SAH. Indeed, in the systematic review conducted by de Rooij *et al*., only 7 out of 58 studies did use necropsy [1]. Our study therefore remains comparable to the majority of epidemiological studies on SAH.

## Conclusions

World age-standardized SAH incidence in Martinique is 3.29 per 100 000 person-years (CI 95% 1.74–4.84) which is low compared to rates observed in other countries. Incidence rate on the island is however similar to neighboring Barbados (WI). This low rate is possibly related to environmental factors and most particularly to a low rate of smoking in the Martinican population. For now, the role of these risk factors on observed low SAH incidence in Martinique remains unclear and further studies are needed in order to counter the paucity of information and to implement evidence-based management of SAH in Martinique.
